# Study of the effect of zinc oxide nanoparticles coated with salicylic acid on intra-abdominal adhesions in rats

**DOI:** 10.1186/s12876-025-04391-z

**Published:** 2025-11-10

**Authors:** Mohammadreza Aghababaei Ziarati, Leila Zarei, Omid Rajabzadeh, Abdolrazagh Marzban, Abbas Raisi, Zahra Haghighatian, Mohammad Kazem Shahmoradi

**Affiliations:** 1 Department of Virology, School of Medicine, Lorestan University of Medical Sciences, Khorramabad, Iran; 2 Palliative Care Reasearch Center, Ur.C., Islamic Azad University, Urmia, Iran; 3 Razi Herbal Medicines Research Center, Lorestan University of Medical Sciences, Khorramabad, Iran; 4 Department of Clinical Sciences, Faculty of Veterinary Medicine, Lorestan University, Khorramabad, Iran; 5 Department of Pathology, School of Medicine, Lorestan University of Medical Sciences, Khorramabad, Iran; 6 Department of General Surgical, Faculty of Medicine, Lorestan University of Medical Sciences, Khorramabad, Iran

**Keywords:** Intra-abdominal adhesion, Salicylic acid, Zinc oxide nanoparticles

## Abstract

**Objective:**

Peritoneal adhesion is a complication following abdominal surgery, observed in about 66% of cases. Adhesions in the intestines are common, and although they may not cause problems, they can sometimes lead to intestinal obstruction. In this study, zinc oxide nanoparticles coated with salicylic acid were used for the first time to prevent the formation of abdominal adhesions after surgery in rats.

**Methods:**

In this study, 40 rats were evaluated in four groups (sham group (laparotomy without abdominal washing), control group (laparotomy and abdominal washing with normal saline), salicylic acid intervention group 27%, and salicylic acid intervention group 54%) regarding the severity of intra-abdominal adhesions after laparotomy. The evaluation of intra-abdominal adhesions was conducted through four criteria (clinical findings, macroscopic findings, histopathological findings, and ELISA findings).

**Results:**

Both intervention groups (27% and 54% salicylic acid–coated ZnO nanoparticles) showed a statistically significant reduction in adhesion severity compared to the control group (*P* < 0.05) across all assessment modalities (macroscopic, histopathological, and ELISA). However, no significant difference was observed between the two intervention doses (*P* > 0.05).

**Conclusion:**

The present study demonstrated that washing the abdominal cavity with 27% and 54% salicylic acid–coated ZnO nanoparticles can significantly reduce the risk of postoperative abdominal adhesions.

## Introduction

Peritoneal adhesion is a common complication following abdominal surgery, occurring in approximately 66% of cases [1]. The highest risk is associated with intestinal surgeries, particularly colorectal procedures, and adhesions may develop within the first year postoperatively [1]. Clinically, they can lead to chronic abdominal or pelvic pain, small bowel obstruction, and female infertility [2]. Statistics have shown that nearly 1% of individuals with peritoneal adhesions require hospitalization [3]. The pathogenesis begins with peritoneal injury, which triggers an inflammatory response mediated by immune cells such as mast cells and macrophages. These cells release histamine and serotonin, leading to vasodilation, increased vascular permeability, and leakage of fibrinogen-rich plasma into the peritoneal cavity [4]. This process is accompanied by the migration of monocytes, plasma cells, polymorphonuclear cells, and histiocytes to the surgical site [5, 6]. Tissue damage activates the coagulation cascade, resulting in thrombin-mediated conversion of fibrinogen to fibrin, which deposits on the peritoneal surface and, if not lysed, undergoes fibroblast proliferation and collagen deposition, ultimately forming permanent adhesions.

One of the major consequences of peritoneal adhesions in infertile women is obstruction of the ovulatory pathway due to adhesions between the ovaries, fallopian tubes, or pelvic wall [7]. The most significant clinical manifestation is chronic and debilitating pelvic pain, caused by tension on nerve-rich tissues due to fibrous bands connecting pelvic organs [3, 7]. To minimize adhesion formation during surgery, several principles are recommended: precise hemostasis, tissue hydration, minimal manipulation, use of non-reactive sutures, and avoidance of foreign bodies [3]. Additionally, agents that reduce fibrin deposition or enhance fibrinolysis—such as anticoagulants, anti-inflammatory drugs, antioxidants, and barrier membranes—have been employed [8]. However, due to the multifactorial nature of adhesion formation, no universally effective preventive strategy exists [3, 8].

Recent advances in nanotechnology have introduced novel therapeutic platforms. Various nanoparticle formulations—including polymeric, lipid-based, metallic, and carbon-based systems—have been explored in biomedical applications [9]. Among them, zinc oxide nanoparticles (ZnO NPs) exhibit a broad spectrum of biological activities, including antimicrobial, anti-inflammatory, anticoagulant, and anticancer effects [10]. Today, salicylic acid derivatives—particularly acetylsalicylic acid (aspirin)—are well recognized for their anti-inflammatory and anticoagulant properties. Coating ZnO NPs with salicylic acid is therefore expected to enhance therapeutic efficacy while potentially modulating nanoparticle toxicity. To the best of our knowledge, this is the first study to evaluate the effect of salicylic acid–coated zinc oxide nanoparticles on the prevention of postoperative intra-abdominal adhesions in a rat model. By simultaneously targeting inflammation, oxidative stress, and fibrogenesis—key drivers of adhesion formation—this nanoformulation represents a novel and potentially translatable strategy to address a major surgical complication that significantly impacts patient morbidity, healthcare utilization, and quality of life.

## Materials and methods

### Study design

Rats were randomly assigned to four groups (*n* = 10/group) using computer-generated random numbers (SPSS v.27). Allocation was concealed in sealed envelopes until group assignment: 1- Sham group: In the sham group (*n* = 8), no surgical procedure was done. This group was designed so that we could evaluate the baseline values for ELISA. 2- Control group: Rats in this group were treated with normal saline (Group A), 3- treatment with 27% acetylsalicylic acid (Group B), and 4- treatment with 54% acetylsalicylic acid (Group C). Forty male Wistar albino rats (age: 10–12 weeks; weight: 250–300 g) were obtained from the Laboratory Animal Breeding Center of Lorestan University of Medical Sciences (Khorramabad, Iran). Only males were used to eliminate hormonal variability. Rats were housed in polycarbonate cages (2–3 per cage) under controlled conditions (12-h light/dark cycle, 23 ± 0.2 °C, 55–70% humidity) with ad libitum access to standard rodent chow and water. The present work has been reported in accordance with the ARRIVE guidelines (Animals in Research: Reporting in Vivo Experiments) [11]. Rats with signs of systemic illness, surgical complications (e.g., wound dehiscence, infection), or mortality during the 14-day postoperative period were excluded from analysis. Outcome assessors (macroscopic, histopathological, and ELISA analyses) were blinded to group allocation. Sample size was calculated based on a pilot study (*n* = 5/group) using G*Power v3.1. Assuming α = 0.05, power = 80%, and effect size f = 0.4, a minimum of 8 rats per group was required. We used *n* = 10/group to account for potential attrition.

### Surgical method

After anesthetizing the rats with a combination of ketamine and xylazine (80 mg/Kg, 10 mg/Kg), the abdominal wall was opened, and three longitudinal and transverse incisions, each 2 centimeters long, were made on the peritoneal surface with dimensions of 1 × 1 centimeter on the left side of the abdominal wall. Subsequently, in the control group, 3 cc of normal saline was administered, while in Groups B and C, respectively, 3 cc of zinc oxide nanoparticle solution (dissolved in normal saline) with 27% and 54% acetylsalicylic acid was poured into the abdominal cavity, and the abdomen was closed. Due to the surgery being performed under sterile conditions, the rats did not receive any antibiotics. After 14 days, a repeat laparotomy was performed to evaluate the adhesions that had formed. All rats received subcutaneous meloxicam (1 mg/kg) immediately post-surgery and once daily for 3 days thereafter for analgesia. No additional medications were administered during the 14-day recovery period. Euthanasia was performed using compressed CO₂ gas (99.9% purity, sourced from a medical-grade cylinder) delivered at a flow rate of 20% chamber volume per minute. Rats were monitored until cessation of respiration and heartbeat. Death was ensured by cervical dislocation prior to tissue harvesting.

### Synthesis of zinc oxide nanoparticles coated with salicylic acid

Zinc oxide nanoparticles were synthesized using the hydrothermal method in the presence of salicylate. To this end, 50 milliliters of a solution containing 4.5 g per liter of zinc nitrate hexahydrate was placed on a magnetic stirrer and the temperature was set to 60 degrees Celsius. Then, a hydroalcoholic solution of salicylate (60% ethanol) at concentrations of 0.5 to 1.5% (50 milliliters) was slowly added under stirring conditions. After that, a sodium hydroxide solution (1 molar) was added dropwise to the mixture and it was stirred for 6 h. Finally, the formation of a white suspension indicated the formation of the desired nanoparticles [12].

### Analysis of the formed nanoparticles

After the formation of the nanoparticles, Ultraviolet (UV-Visible) spectroscopy was performed with a spectrophotometer to confirm their formation.

### Determination of therapeutic dose and cytotoxicity of zinc oxide – salicylate nanoparticles

In order to achieve the therapeutic dose of the nanoparticles, their cytotoxicity must first be evaluated in vitro. For this purpose, the cytotoxicity of the nanoparticles was assessed on the hematocrit (HCT)−116 colon cell line [13]. The 3-(4,5-dimethylthiazol-2-yl)−2,5-diphenyltetrazolium bromide (MTT) assay was used according to the protocol, and the (Inhibitory concentration) IC-50 concentration for each group was determined.

### Macroscopic evaluations

After 14 days, adhesions formed were graded based on the number of adhesion bands [14] during a repeat laparotomy as follows:

- Grade zero: No adhesions.
- Grade one: Between viscera or visceral organs and the abdominal wall (one band).
- Grade two: Between viscera or visceral organs and the abdominal wall (two bands).
- Grade three: Between viscera or visceral organs and the abdominal wall (more than two bands) or multiple intestinal adhesions without adhesion to the abdominal wall.
- Grade four: Viscera directly adhered to the abdominal wall (regardless of the number and size of bands).

### Enzyme-Linked Immunosorbent Assay (ELISA) evaluations

The expression of inflammatory factors including Interleukin-6 (IL-6), Interleukin-1 beta (IL-1B), Tumor necrosis factor α (TNF-α), Transforming Growth Factor Beta 1 (TGF-β1), and Vascular endothelial growth factor (VEGF) were examined using commercially enzyme-linked immunosorbent assay (ELISA) kits [15]. The IL-6 ELISA kit was obtained from Elabscience Biotechnology Inc. (Houston, TX, USA), the IL-1β kit from R&D Systems, a Bio-Techne brand (Minneapolis, MN, USA), the TNF-α kit from Thermo Fisher Scientific, Invitrogen brand (Waltham, MA, USA), the TGF-β1 kit from Abcam (Cambridge, UK), and the VEGF kit from MyBioSource Inc. (San Diego, CA, USA). Samples were collected under sterile conditions and washed with cold phosphate-buffered saline (PBS) for removing blood and debris. Tissues were then homogenized in ice-cold RIPA lysis buffer containing protease inhibitors. Homogenates were centrifuged at 12,000 × g for 15 min at 4 °C, and the supernatants were taken. Total protein concentration in each sample was measured using a bicinchoninic acid (BCA) assay. ELISA assays were employed based on the protocols provided in each kit.

### Histopathological evaluations

For histopathological evaluations, tissue samples including peritoneal adhesions and underlying tissue (abdominal muscles) were fixed in 10% neutral buffered formalin [16]. Tissue staining was performed using the Hematoxylin and Eosin (H&E) method according to standard protocols. Finally, based on tissue staining, the H&E slides were evaluated by a pathologist according to the microscopic adhesion classification [17] as follows:

- Based on the severity of the inflammatory response:
  Score 1: Mild inflammatory response. Scattered inflammatory cells (primarily lymphocytes or macrophages) with minimal tissue involvement.
  Score 2: Moderate inflammatory response. Localized areas of dense inflammatory cell infiltration involving < 50% of the tissue section.
  Score 3: Severe inflammatory response. Diffuse, dense inflammatory infiltrate involving > 50% of the examined tissue section, often with edema or tissue destruction.
- Based on granulation:
  Grade zero: No fibroblast proliferation and angiogenesis.
  Grade One: Mild granulation tissue formation involving 1–10.99% of the tissue area.
  Grade Two: Moderate granulation tissue formation involving 11–50.99% of the tissue area.
  Grade Three: Extensive granulation tissue formation involving 51–100% of the tissue area.

### Statistical methods

For statistical analysis of the data, IBM SPSS v.27 software was used. The distribution of groups was analyzed using the one-sample Kolmogorov-Smirnov test. Data with a normal distribution were expressed as mean ± standard deviation (SD) and analyzed using one-way ANOVA, and in the presence of statistical disturbance, the Tukey-Kramer post-hoc test was applied. Data with a non-normal distribution were reported as median and interquartile range. For comparing groups with a non-normal distribution, the non-parametric Kruskal-Wallis test was used followed by Dunn’s Test as post hoc. The significance threshold was set at *P* < 0.05. Qualitative data related to histopathological parameters were analyzed using the chi-square test.

## Results

All samples in this study were similar in terms of nutrition and activity before surgery, and no issues were observed during or after the procedure. All samples tolerated anesthesia and surgery and returned from anesthesia without complications. None of the samples exhibited symptoms such as redness, swelling, watery bloody discharge, or infection.

**Fig. 1:**
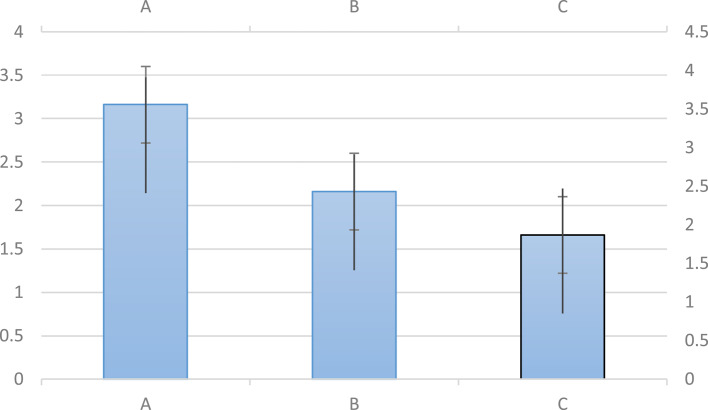
Results of macroscopic examinations. Adhesion intensity based on the Nair scale

**Fig. 2:**
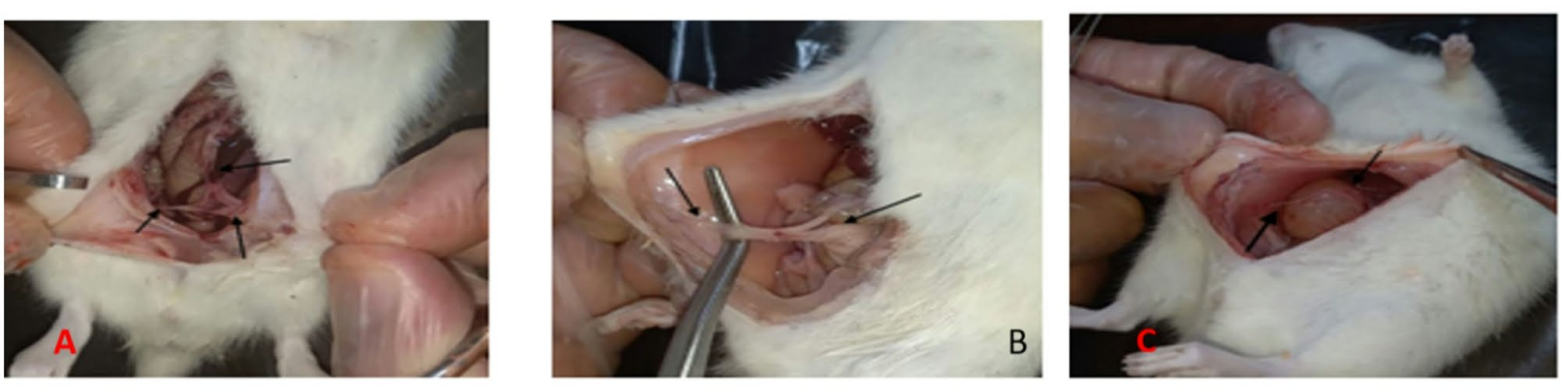
Adhesions. **A** Control group (**A**), **B** Intervention group with 27% salicylic acid (**B**), **C** Intervention group with 54% salicylic acid (**C**), Arrows indicate the bands formed

After performing laparotomy under identical conditions, we found that both treatment groups (B, C) had a statistically significant difference in reducing adhesion levels in the macroscopic examination compared to the control group (A) (*P* < 0.05). However, no statistically significant difference was observed between the treatment groups with 27% acetylsalicylic acid (group B) and 54% acetylsalicylic acid (group C) (*P* > 0.05) as shown in Figs. 1 and 2.

### Histopathological findings

The examination of the histopathology results of Hematoxylin and Eosin (H&E) staining based on Table 1 in the different study groups yielded the following results.

**Table 1:** Histopathological findings

Groups	Fibroblast proliferation and collagen deposition	Infiltration of chronic inflammatory cells	Edema and congestion
Sham	0	0	0
Group A (normal saline)	+ 2.6	+ 2.4	+ 2.8
Group B (27% acetylsalicylic acid)	+ 1.6	+ 1.6	+ 1.4
Group C (54% acetylsalicylic acid)	+ 1.2	+ 1.2	+ 1.2

**Fig. 3:**
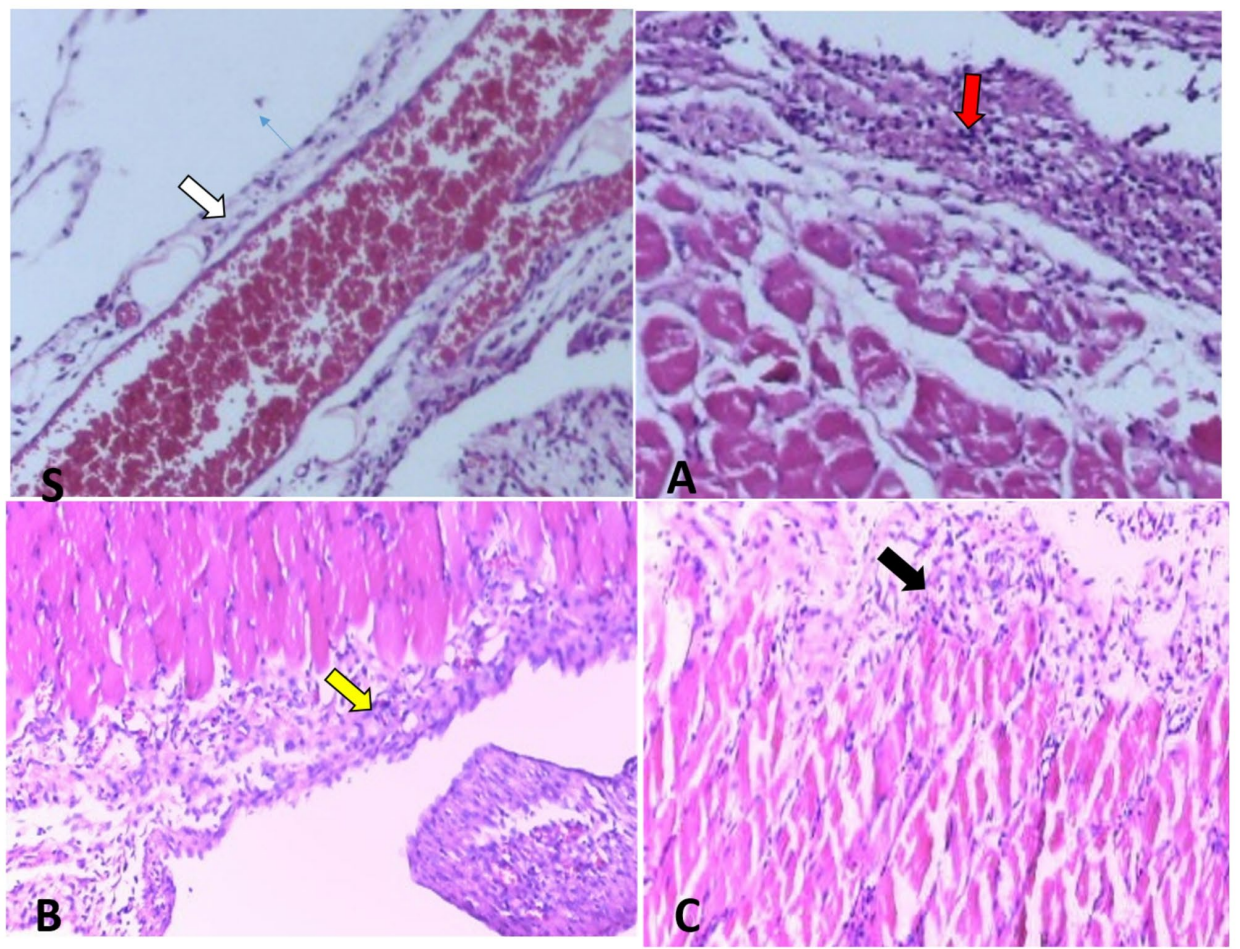
Histopathology images stained with H&E. Red arrow in group **A** shows very severe levels of inflammation, collagen deposition, and congestion; yellow and black arrows in groups **B** and **C** shows lower levels of inflammation, collagen deposition, and congestion respectively. S: Sham, **A**: group **A**, **B**: group **B**, **C**: group **C**

**Fig. 4:**
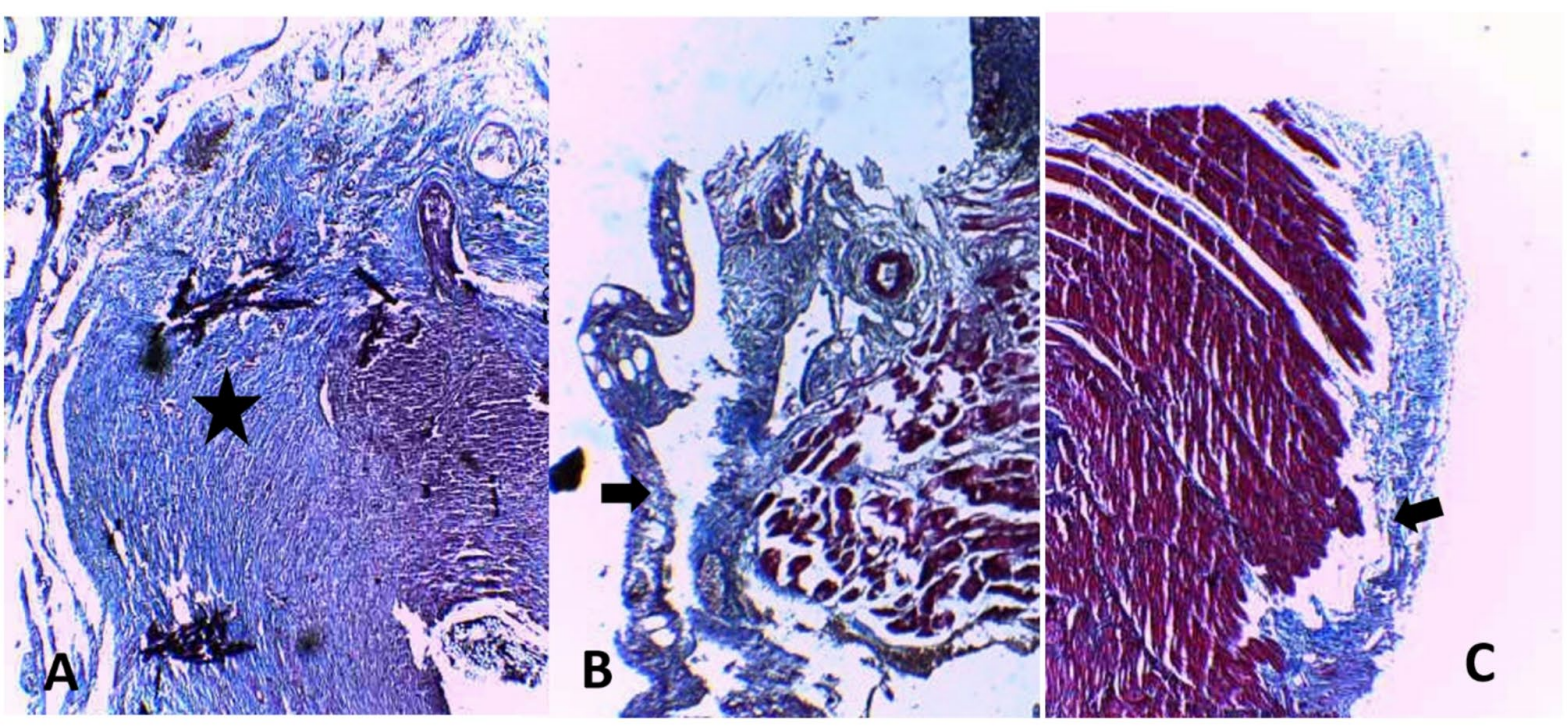
Histopathology images stained with trichrome. **A** Control group, **B** Intervention group 27%, **C** Intervention group 54%. Black star indicates sever fibrosis, black arrows in groups **B** and **C** show decreasing fibrosis in peritoneal epithelium

In the conducted investigations, there was a statistically significant difference in fibroblast proliferation and collagen deposition between the control group (A) and the intervention groups (B, C) (*P* < 0.05) (Table 1), such that fibroblast proliferation and collagen deposition were more pronounced in the control group. However, no statistically significant difference was observed between the two intervention groups (*P* > 0.05). Regarding the infiltration of chronic inflammatory cells, a statistically significant difference was found between the control group (A) and the intervention groups (B, C) (*P* < 0.05), with chronic inflammatory cell infiltration being more severe in the control group. Again, no statistically significant difference was noted between the two intervention groups (*P* > 0.05). In terms of edema and tissue congestion (Figs. 3 and 4), there was a statistically significant difference between the control group (A) and the intervention groups (B, C) (*P* < 0.05), indicating that edema and tissue congestion were more severe in the control group. However, no statistically significant difference was observed between the two intervention groups (*P* > 0.05).

### ELISA findings

The results of ELISA for the parameters TNF-α, TGF-β, IL-6, IL-1, and VEGF in the various tested groups were observed as follows (Table 2).

**Table 2:** ELISA findings

*P*-Value	Experimental groups				Parameters
	Group C	Group B	Group A	sham	
0.0001>	52.59 ± 11.76	81.09 ± 8.98	137.44 ± 20.8	27.54 ± 5.36	**TNF-α (ng/L)**
0.0001>	114.48 ± 7.95	169.82 ± 11.11	284.1 ± 31.3	95.73 ± 6.8	**TGF-β (ng/L)**
0.0001>	67.26 ± 3.71	87.09 ± 9.65	109.71 ± 6.31	49.31 ± 3.99	**IL-6 (ng/L)**
0.0001>	20.29 ± 3.12	28.24 ± 2.83	39.37 ± 3.13	14.61 ± 4.07	**IL-1 (ng/L)**
0.0001>	12.753 ± 1.967	19.025 ± 1.05	38.425 ± 2.187	7.641 ± 1.656	**VEGF (ng/L)**

A significant difference was observed in TNF-α among the experimental groups (*P* < 0.0001). The control group showed a significant increase compared to the sham group (*P* < 0.0001). The Salicylic acid (54%) group showed a significant decrease compared to the control group (*P* < 0.0001). The sham group had a significant decrease compared to the Salicylic acid (27%) group (*P* < 0.0001) and the Salicylic acid (54%) group (*P* = 0.003). The Salicylic acid (54%) group showed a significant decrease compared to the Salicylic acid (27%) group (*P* = 0.001).

There was a significant difference in TGF-β among the experimental groups (*P* < 0.0001). The control group showed a significant increase compared to the sham group (*P* < 0.0001). The Salicylic acid (54%) group exhibited a significant decrease compared to the control group (*P* < 0.0001). The Salicylic acid (27%) group also showed a significant decrease compared to the control group (*P* < 0.0001). The sham group had a significant decrease compared to the Salicylic acid (27%) group (*P* < 0.0001) and the Salicylic acid (54%) group (*P* = 0.001). The Salicylic acid (54%) group demonstrated a significant decrease compared to the Salicylic acid (27%) group (*P* = 0.001).

In IL-6, a significant difference was observed among the experimental groups (*P* < 0.0001). The control group showed a significant increase compared to the sham group (*P* < 0.0001). The Salicylic acid (54%) group exhibited a significant decrease compared to the control group (*P* < 0.0001). The Salicylic acid (27%) group also showed a significant decrease compared to the control group (*P* = 0.001). The sham group had a significant decrease compared to the Salicylic acid (27%) group (*P* < 0.0001) and the Salicylic acid (54%) group (*P* < 0.0001). The Salicylic acid (54%) group demonstrated a significant decrease compared to the Salicylic acid (27%) group (*P* = 0.002). Significant differences were observed in IL-1 among the experimental groups (*P* < 0.0001). The control group showed a significant increase compared to the sham group (*P* < 0.0001). The Salicylic acid (54%) group demonstrated a significant decrease compared to the control group (*P* < 0.0001). The Salicylic acid (27%) group also showed a significant decrease compared to the control group (*P* < 0.0001). The sham group exhibited a significant decrease compared to the Salicylic acid (27%) group (*P* < 0.0001) and the Salicylic acid (54%) group (*P* = 0.01). The Salicylic acid (54%) group showed a significant decrease compared to the Salicylic acid (27%) group (*P* < 0.0001). In VEGF, significant differences were observed among the experimental groups (*P* < 0.0001). The control group showed a significant increase compared to the sham group (*P* < 0.0001). The Salicylic acid (54%) group demonstrated a significant decrease compared to the control group (*P* < 0.0001). The Salicylic acid (27%) group also showed a significant decrease compared to the control group (*P* < 0.0001). The sham group exhibited a significant decrease compared to the Salicylic acid (27%) group (*P* < 0.0001) and the Salicylic acid (54%) group (*P* < 0.0001). The Salicylic acid (54%) group showed a significant decrease compared to the Salicylic acid (27%) group (*P* < 0.0001).

## Discussion

The present research is an experimental study aimed at investigating the macroscopic and microscopic effects of zinc oxide nanoparticles coated with salicylic acid on intra-abdominal adhesions following surgery in rats. Intra-abdominal adhesions account for the highest number of complications after surgery and may represent one of the largest unresolved issues in modern medicine. Epidemiological studies describe the formation of adhesions post-surgery in 50–100% of surgical patients. They are responsible for 65 to 75% of small bowel obstructions, which can be life-threatening and require subsequent surgical intervention [3]. Additionally, 15–20% of female infertility is attributed to intra-abdominal adhesions, with pelvic adhesions being the most common morphological changes observed in women suffering from chronic abdominal pain. Therefore, intra-abdominal adhesions have a significant impact on the quality of life for many patients.

In the present study, the evaluation of intra-abdominal adhesions was conducted through four criteria (clinical findings, macroscopic findings, histopathological findings, and ELISA findings). The mice were clinically similar prior to the initial laparotomy. After the intervention, all samples tolerated anesthesia and surgery, and no differences were observed among them regarding nutritional status, wound condition, and clinical signs. Following the re-laparotomy on the samples, the macroscopic condition of the adhesions formed was assessed using the scale described by Nair et al. There was a statistically significant difference in the severity of adhesions between the intervention groups (B, C) and the control group (A), with the severity of adhesions being higher in the control group (A). No statistically significant difference in the severity of intra-abdominal adhesions was observed between the two intervention groups (abdominal lavage with 27% and 54% salicylate). Numerous studies have been conducted using various materials regarding the prevention of peritoneal adhesions. Almamar et al. investigated the systemic effect and absorption rate of intraperitoneal heparin with or without hyaluronic acid in preventing postoperative abdominal adhesions and demonstrated its preventative role [18]. In a study by Legrand et al. comparing the effects of non-steroidal anti-inflammatory drugs and thromboxane inhibitors in a rabbit model for adhesion prevention, the efficacy of these substances in preventing peritoneal adhesions was shown [19]. Chiorescu et al. demonstrated that intraperitoneal administration of rosuvastatin prevents postoperative peritoneal adhesions by reducing the release of tumor necrosis factor [20]. In 2017, Parsa and colleagues investigated the effect of peritoneal lavage with lidocaine and bupivacaine on reducing the formation of abdominal adhesions in rats, demonstrating the efficacy of these agents in decreasing peritoneal adhesion, although they were not effective in preventing the formation of peritoneal adhesions [21]. In a study conducted by Portilla et al., the preventive effect of intraperitoneal injection of vitamin E on peritoneal adhesion was shown [22]. Vitamin E inhibits cyclooxygenase-2 as well as the endogenous conversion of arachidonic acid to Prostaglandin E2 (PGE2) and PGF2, and it reduces inflammation by preventing oxidation initiated by free radicals. The challenge of using various materials to prevent peritoneal adhesions in contemporary research underscores the significance of this topic. Three histopathological findings indicating the severity of adhesions, infiltration of chronic inflammatory cells, and edema and congestion were evaluated in this study. For all three findings, a statistically significant difference was observed between the control group (A) and the intervention groups (B, C), with the severity of all three being higher in the control group compared to the intervention groups. However, no statistically significant difference was noted between the two intervention groups. In the assessment of inflammatory factors in plasma, the results indicated that the plasma levels of these parameters in the control group were significantly higher compared to the intervention groups (B, C). Additionally, when comparing the intervention groups, the plasma levels of all inflammatory parameters in the acetylsalicylate %27 group (B) were significantly higher than in the acetylsalicylate %54 group (C). Localized inflammation is one of the known mechanisms for the formation of abdominal adhesions. Acetylsalicylic acid also possesses recognized anti-inflammatory properties. The present study supports the hypothesis that the suppression of localized inflammation through the use of anti-inflammatory medications, including acetylsalicylic acid, may reduce the risk of abdominal adhesion formation. Olbert and colleagues examined the effects of ketoprofen as an anti-inflammatory drug on abdominal adhesions following surgical procedures, reaching results similar to those of the present study [23]. Similarly, Bayhan and colleagues evaluated the anti-inflammatory effects of pirfenidone, also achieving comparable results [24]. The anti-inflammatory properties of acetylsalicylic acid play a key role in preventing the infiltration of chronic inflammatory cells. Chronic inflammation is characterized by the continuous infiltration of immune cells, including macrophages, lymphocytes, and neutrophils, into affected tissues. These cells release various types of pro-inflammatory cytokines, chemokines, and growth factors, which perpetuate the inflammatory response and tissue damage.

Inflammatory cytokines play a major role in the formation of postoperative adhesions. Pattern recognition receptors (PRRs) on immune cells detect danger signals from pathogen-associated molecular patterns (PAMPs) or damage-associated molecular patterns (DAMPs), which are generated by various endogenous stress signals from the host. The interleukin-1 (IL-1) cytokine family, including IL-1α, IL-1β, IL-18, IL-33, IL-36α, IL-36β, and IL-36γ, acts as DAMPs and stimulates sterile inflammation due to tissue necrosis, while also enhancing inflammation associated with tissue damage from infection. The cyclooxygenase (COX) and 5-lipoxygenase (LOX-5) pathways, metabolized by arachidonic acid (AA), produce numerous pro-inflammatory lipid mediators that are involved in the classic signs of inflammation, including redness, fever, pain, swelling, and loss of function, which are designed to eliminate harmful stimuli. One important family of receptors that stimulate inflammation includes Toll-like receptors (TLRs). TLR4 signaling, mediated by the adaptor MyD88, activates the nuclear factor kappa B (NF-kB) transcription factor, resulting in the expression of genes for pro-inflammatory factors such as tumor necrosis factor (TNF). These proteins play a significant role in inflammatory diseases [3, 5].

Oxidative stress is caused by an imbalance between the production of reactive oxygen species (ROS) and antioxidant defenses, which leads to cell damage. Aspirin uses several mechanisms to modulate oxidative stress. This drug inhibits cyclooxygenase enzymes and reduces the production of pro-inflammatory prostaglandins and thromboxanes [25]. This reduction in inflammation is partially due to reduced ROS production, as inflammatory cells are important sources of ROS. In addition, acetylsalicylate increases the expression and activity of various antioxidant enzymes such as superoxide dismutase (SOD), catalase and glutathione peroxidase (GPx). These enzymes play vital roles in ROS detoxification and maintaining redox balance. SOD catalyzes the dismutation reaction of superoxide radicals to hydrogen peroxide, which is then broken down into water and oxygen by catalase and GPx, thus reducing oxidative damage. In addition, acetyl salicylate induces the expression of the erythroid transfer factor 2 related to the nucleus (Nrf2) ، A factor that regulates the expression of antioxidant response elements (AREs) in the promoter regions of many antioxidant and protective cell genes [26]. This elevated expression increases cellular antioxidant capacity and further protects cells from oxidative damage. However, chronic use of this drug paradoxically can lead to increased oxidative stress in the tissues of the digestive tract, which may cause damage to the mucus and cause scarring. This condition is attributed to the destruction of protective prostaglandins, increased production of ROS in the mitochondria, and disruption of mitochondrial function [27]. Thus, while acetyl salicylate exerts beneficial effects by modulating oxidative stress pathways and strengthening antioxidant defenses، its effect is complex and context-dependent, requiring an accurate balance to maximize therapeutic benefits and minimize side effects. Acetyl salicylate has been shown to express adhesion molecules on endothelial cells such as intercellular adhesion molecule-1 (ICAM-1) and vascular cell adhesion molecule-1 (VCAM-1) for adhesion and leucocytes’ armies are vital to tissues, modulating [28]. By reducing the expression of these adhesion molecules, acetyl salicylate reduces the ability of inflammatory cells to stick to the vascular endothelium and penetrate it, thus further reducing chronic inflammatory cell penetration. Thus, the ability of acetyl salicylate to inhibit collagen cross-linking and reduce chronic inflammatory cell penetration highlights its therapeutic potential in the management of chronic inflammatory diseases and related fibrotic conditions. Acetyl salicylate has significant effects on the cross-linking of collagen and the infusion of chronic inflammatory cells. Collagen cross-linking, a process in which collagen fibers are stabilized through enzymatic and non-anzymic reactions, is essential for maintaining tissue integrity and function. However, excessive or abnormal collagen cross-linking can lead to tissue stiffness and fibrosis, which are common features of chronic inflammatory diseases [29]. Studies have shown that acetyl salicylate inhibits the activity of the enzyme lysyl oxidase (LOX), which is responsible for catalyzing the formation of covalent bonds between collagen molecules. By inhibiting LOX, acetyl salicylate can reduce collagen over-crossing bonds. Immune reactions also play a significant role in the development of abdominal adhesions. Kazemi and colleagues examined the effect of two prednisolones and sirolimus drugs (a macrolide with immunosuppressive properties) on the prevention of abdominal adhesion formation. Prednisolone is both an immunosuppressive and anti-inflammatory. The above study showed that these two drugs, especially when administered together, can greatly reduce the risk of developing abdominal adhesions [30]. Our results showed that in the control group, levels of measured inflammatory cytokines increase after peritoneal adhesion caused by the operation, and both concentrations of acetyl salicylate in treatment groups they reduce TNF-α levels after peritoneal adhesion after surgery, and the above studies show our results in reducing TNF-α anti-inflammatory cytokine, thereby reducing peritoneal adhesion Confirms after the operation. Past studies have shown that increased TGF-β levels are associated with greater adhesion formation [31]. neutralizing TGF-β using antibodies against TGF-β prevents adhesion in mice. The results showed that treatment with acetyl salicylate reduced TGF-β levels [32]. The results of this study are consistent with previous studies. Past studies have shown that VEGF levels increase during adhesion formation [33]. Increasing VEGF levels by increasing the size of endothelial cells to the adhesion site increases fibrin adhesions caused by blood supply formation. Inhibiting VEGF using the monoclonal antibody VEGF reduces peritoneal adhesion after abdominal surgery [34]. Our results showed that acetyl salicylate reduces the VEGF level after peritoneal adhesion caused by the action. Our histopathological findings particularly reduced fibroblast proliferation, collagen deposition, and inflammatory infiltration are consistent with recent advances in anti-adhesion biomaterials. For instance, a multifunctional hernia repair biopatch significantly attenuated fibrosis by modulating TGF-β1 and extracellular matrix remodeling [35], mirroring the antifibrotic effects observed in our ZnO–salicylate groups. Similarly, autologous cytokine-rich serum (ACRS) and PRP treatments in a rat uterine adhesion model reduced inflammation but also stimulated granulation tissue [36], whereas our nanoformulation suppressed both inflammation and fibroblast activity without excessive tissue reaction. Furthermore, our results align with curcumin supplementation studies, which reported decreased intra-abdominal adhesion severity via antioxidant and anti-inflammatory mechanisms [37]. Notably, the nano-delivery of salicylic acid in our study may enhance bioavailability and prolong local anti-inflammatory action compared to free compounds.

## Conclusions

The present study showed the effectiveness of zinc oxide nanoparticles coated with acetyl salicylate in reducing abdominal adhesions after surgery in rat models. The present study showed that washing the abdominal cavity with acetyl salicylate 27% and 54% can significantly reduce the risk of developing abdominal adhesions following surgery. However, there was no significant difference between the two different doses of acetyl salicylate. This study could be a starting point for further research and the development of new nanotechnology-based therapies that have the potential to significantly reduce problems and complications after surgery and improve quality of life They have patients.

### Strengths and limitations

Strengths include comprehensive multi-modal assessment (macroscopic, histopathological, and molecular) and adherence to ARRIVE guidelines. Limitations include: (1) short-term follow-up (14 days), precluding assessment of chronic adhesion evolution; (2) use of only male rats, limiting generalizability to females; (3) inability to isolate individual contributions of ZnO vs. salicylic acid; and (4) lack of biodistribution data for nanoparticles.

Suggestions for future studies:

- Longer-term in vivo assessments (e.g., 4–8 weeks) to evaluate adhesion durability and organ safety.
- Comparative arms testing ZnO alone, salicylic acid alone, and the coated formulation to deconvolute mechanisms.
- Biodistribution and clearance studies of the nanoparticles.
- Exploration of alternative delivery systems (e.g., hydrogels or sprays) for clinical translation.

## Data Availability

The datasets used and/or analyzed during the current study available from the corresponding author upon request.

## Abbreviations

UV: Ultraviolet
HCT-116: Human colon cancer cell line
MTT: 3-(4, 5-dimethylthiazolyl-2)-2, 5-diphenyltetrazolium bromide) assay
IL: Interleukin
TNF: Tumor necrosis factor
TGF: Transforming Growth Factor
VEGF: Vascular endothelial growth factor
H&E: Hematoxylin and eosin
PGE: Prostaglandin E
PGF: Prostaglandin F
